# Technology enhanced health professions education (TEHPE) in Eastern and Southern Africa: a Needs Assessment

**DOI:** 10.21203/rs.3.rs-5890423/v1

**Published:** 2025-02-10

**Authors:** Shalote Rudo Chipamaunga, Detlef Prozesky, Elliot Kafumukache, Patricia Katowa-Mukwato, Kefalotse Dithole, Lynette van der Merwe, Samuel Owusu-Sekyere, Mmoloki Cornelius Molwantwa, Rudo Gwini

**Affiliations:** University of Zimbabwe; University of Botswana; University of Zambia; University of Zambia; University of Botswana; University of the Free State; Kwame Nkrumah University of Science and Technology; University of Botswana; National University of Science and Technology

**Keywords:** Educational technology, Fourth Industrial Revolution, SAMR Model, undergraduate health professions education

## Abstract

**Background:**

The COVID-19 pandemic escalated the adoption and development of technology-enhanced health professions education during large-scale lockdown mandated in multiple countries. Although the use of technology is a hallmark of the education of healthcare professionals, including clinical education, challenges including poor availability, lack of skills and support for technology-enhanced learning and teaching are reported. This study aimed to assess the needs for technology enhanced health professions education in higher education institutions in Eastern and Southern Africa.

**Methods:**

This was a descriptive, cross-sectional survey to yield quantitative and qualitative data from healthcare students, educators and managers. The Substitution, Augmentation, Modification, Redefinition Model, our conceptual framework, underpinned our study and served as an organizing framework.

**Results:**

Twelve program directors, 35 educators and 264 students responded from undergraduate nursing and medical programs. The findings indicate that ‘Other technologies used’ were relatively few – only 89 instances were mentioned by 299 respondents, and of these 89 some were basic hardware (laptops, data projectors) and online data source, which could fall under one or more of the 13 types that were drawn from the literature and the individual experiences of the researchers. There was extreme variation (between programmes and institutions) in the use of technologies. This finding is best explained by variations in the way in which programmes are offered and ‘educator’ preferences – there is clearly no ‘one size fits all’. On reasons for using educational technology, there is wide variation between categories of respondent, programmes and institutions. The main obstacles to the use of technology include that staff and students lack the required skills; no training is available; poor connectivity; lack of hardware and of funds to buy software; and lack of online learning resources.

**Conclusions:**

The information generated, in such detail was not available before and opens many opportunities for further research as well as for planning and implementing improvements in technology enhanced health professions education. Based on the data, we propose practical recommendations organized around: Infrastructure Development, Training and Capacity Building, Curriculum reviews to align with technology, Institutional Support and Policy Development, Collaboration and Resource Sharing and Addressing Equity and Inclusion.

## BACKGROUND

Education is an integral pillar of any country’s sustainable growth and development [1]. Higher education institutions are essential to a sustainable society’s growth and development and should function as transforming forces [2,3]. Regarding Sustainable Development Goals, Kasinathan et. al observed that technology has significant potential to drive humanity into sustainable development [4]. The world also now finds itself in the Fourth Industrial Revolution, also called Industry 4.0 [5,6]. As has been the case with the previous industrial revolutions, education is changing to accommodate the needs of the new 4IR. This new system or set of educational processes has been termed Education 4.0. It aims to develop the competencies and skills that workers in the 4IR require, through applying active learning, technology-based learning and student-centred learning [7,8].

More recently Education 5.0 has arisen as a new concept [9]. It also involves the use of new technologies to transform education, but in addition emphasizes the need for a constructivist, humanistic and andragogical orientation poised for deep learning [4,10,11].

Some higher education institutions have been influenced by the ‘Education 5.0’ movement which is highly rated in terms of its association with current business needs and integration with industrial and technological developments [12,13]. Education 5.0 functions within Society 5.0 which is regarded as a super-intelligent society that promotes the convergence of cyberspace and physical space and focuses on human-oriented solutions and social innovation. The expectation of such a combination is to develop an environment where humans and robots with artificial intelligence (AI) coexist and work to improve the quality of human life. A further development in the field of technology for education is the advent of disruptive technology like artificial intelligence (AI) software and social media which are already being used by Educators and students worldwide [16]. Rowan and Casey suggest a Triple Helix ‘Academia-Industry-Authority’ approach to creating and sharing disruptive tools for addressing emerging needs for development [17]. Advances in industrialization require technology to enable adaptation to complex systems in the health sciences.

Against this background, the use of technology in its various forms has long been a feature of the education and training of health professionals [1,18]. Worldwide a plethora of opportunities for using technology in such training have been developed and used, including augmented and virtual reality platforms that are available for use in modern education [19]. In clinical education the process of acquiring knowledge and skills needs to be more experiential, self-directed, and hands-on than in many other disciplines. However virtual or mixed reality interventions are becoming more accessible, and can be used in an integrated way with current, more traditional learning systems [20]. Rath, Satpathy and Patnaik point out that augmented reality (AR) and virtual reality (VR) technologies are already having a major influence on medicine and health care, the implication being that students need to be exposed to important skills in these areas [21].

Even before the COVID-19 pandemic struck, those institutions that had not taken serious measures to migrate to a higher level of use of technology would have been aware that they were lagging behind the requirements of modern educational approaches. In the situation created by the pandemic students were unable to attend their usual learning events and yet had to move forward in their training. There is good evidence that the advent of large-scale lockdown national policies resulting from the COVID-19 pandemic stimulated the development of technology-enhanced health professions education (TEHPE) [22,23]. However, a study by Jeffries et al. found that while COVID-19 resulted in transition to more online HPE the change was subsequently unstable since the change to technology was not always widely accepted, was marred by inequities (socio-economic, gender and geographical) and was not subject to ongoing evaluation to indicate where improvements were needed [24]. Besides the lack of opportunities for essential learning in practical settings, the large-scale use of online learning during the pandemic also had ethical consequences: ‘education with basic humanity’ was neglected and professionalism, interpersonal relationships, mentoring and communication skills suffered; this trend may be continuing [25].

Challenges to expanding the use to technology in educating health professionals have also been discussed. Challenges such as unavailability, lack of skills leading to resistance, lack of support that curtail the adoption and use of technology have been identified and some solutions have been outlined [18,26]. While acknowledging the empowering possibility of technology use in education in Africa, DeFrank-Cole stresses the reality of the digital divide, between countries, within countries and relating to gender [27] . There is often a lack of connectivity, computers and other devices – let alone the more sophisticated technologies listed in the previous paragraph – and digital literacy is also often insufficient. Even electricity supply has been and may still be a challenge [29]. An in-depth investigation of e-learning in sub-Saharan Africa and low-resource settings by Barteit et al. makes sobering reading [30]. They conclude that e-learning for medical education was not doing justice to medical students in these settings; that short, one-off projects were common (so-called ‘cul-de-sac pilots’); and that ‘technological development has overwhelmed rather than revolutionized medical education’. Both DeFrank-Cole and Barteit et al. stress the need for sustained investment in integrating technology into existing platforms [27,30]. There are also legitimate doubts about the effectiveness of the use of technology in training health professionals. In a large-scale Indian study by Hirkani & Supe, the evidence was mixed even though students had a positive attitude towards it [31]. The authors conclude that more evidence needs to be generated regarding its effectiveness, so that future decisions can be based on evidence. In Pakistan Sadiq et al. report a broadly successful project to empower educators in greater use of technology in their teaching, but also noted educators’ limited e-learning skills and failure to engage with the new technologies, as well as technical hurdles encountered [32].Granger, Liu & Gladman have also found that the grand vision of Education 4.0 is in danger of being watered down by technology mostly being used for lower level pedagogical changes (substitution and augmentation) in the SAMR model [33,34]. In this regard a study from Uganda revealed low uptake of TEHPE activities after ten years of effort, again with lower-level pedagogical changes. The empirical evidence gathered in the study led to recommendations to create an eLearning Unit, to improve skills and knowledge in educational technologies, to expand infrastructure for TEHPE and to design and implement an educational technology policy [35].

From the available evidence reviewed, there is clear evidence that TEHPE is thoroughly in place in the world, and that this trend is likely to have intensified during the COVID-19 pandemic. Interestingly however there is little information available about the extent to which TEHPE is currently being used in the training of health professionals specifically in Eastern and Southern Africa. It is also not known whether the sudden spurt in TEHPE during the COVID-19 pandemic has been maintained, nor is it known whether what has been retained is of a quality that deserves its retention. The obstacles impeding its use and the factors facilitating its use in this geographically specific setting are similarly not known.

### Conceptual framework

**Figure 1:**
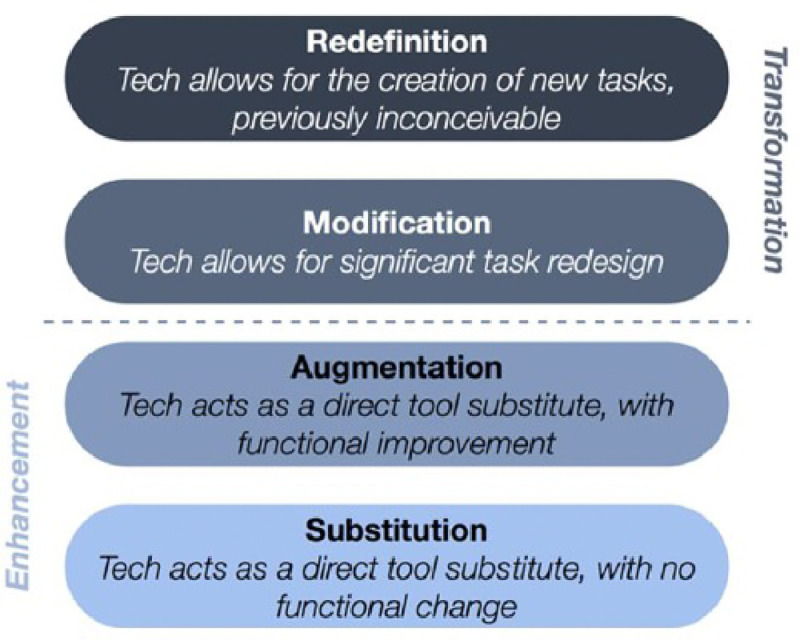
The SAMR (Substitution, Augmentation, Modification, Redefinition) Model

The SAMR (Substitution, Augmentation, Modification, Redefinition) Model underpins our study and serves as an organizing framework for the different types of technology in current use in the institutions under study [36]. The SAMR model is a tool which provides guidance in describing and categorizing uses of educational technology in the classroom. The model is intended to guide educators to enhance their teaching and learning through the adoption, adaptation or transformation of educational approaches using technology (see Figure 1 below).

To demonstrate our conceptual understanding and application of SAMR, we describe each level briefly and include hypothetical examples of each [34,36]. There are two dimensions in the model, enhancement, and transformation, and two levels in each dimension.

### **Enhancement** – Substitution and Augmentation:

At the *Substitution* level, digital technology is substituted for analog technology, but the substitution generates no functional change [36,37]. For example, an educator may substitute hard copy handouts with electronic versions. At the *Augmentation* level, technology is exchanged, and the function of the task or tool positively changes in some way. An example is when an educator adds links to electronic versions of learning material. Larger volumes of electronic material like books and videos are examples.

### **Transformation** – Modification and Redefinition

At the level of *Modification*, technology enables significant task redesign. Examples of this include the recording of lectures, podcasts etc. which are textual, visual, audio tools for construction of knowledge which can be shared across groups of students. At *Redefinition*, technology enables the creation of new tasks that were previously inconceivable like tools for visualization of narrative and structural aspects of text. Examples of this include artificial intelligence (AI) and use of software for data analysis.

## METHODS

### Design

This was a descriptive, cross-sectional survey to yield quantitative and qualitative data that address our research objectives as outlined above. We built an analytic element to compare data according to the nature of the variables obtained. This enabled us to determine the differences in the technology used between programmes and countries and the needs for enhancement of the technology

### Objectives

Relating to the education of health professions in higher education institutions offering health sciences programmes the objectives of the proposed study are to:

1. Determine the types of educational technology being used
2. Identify the frequency with which different technologies are being used
3. Reveal the reasons why these technologies are being used
4. Determine teacher and student opinions about the usefulness of the technologies
5. Identify obstacles to the use of these technologies
6. Verify factors facilitating the use of these technologies

### Definitions and data to be collected

Tables 1 and 2 present the definition of terms and data to be collected respectively.

Concerning objective no. 6 ‘Verify factors facilitating the use of these technologies’: When preparing the data collection tools it became clear that factorsfacilitating the use of technology are in almost all cases the opposite of the obstacles encountered. It was decided therefore not to collect data about thefacilitating factors.

### Study population and sampling

The study population was healthcare professionals who are also educators in health professions education programmes in selected Eastern and Southern African higher institutions of learning. Given the short implementation timeframe for this study, it will be feasible to focus only on educators and students in undergraduate medical and nursing programmes in PACoTEH member countries as the study population. These are summarized in Table 3.

#### Inclusion criteria

Programmes in Eastern and Southern African countries which:

- Train doctors and nurses
- Offer bachelor’s degree level qualifications in medical and nursing programmes
- Are recognized by national professional bodies and/or government structures
- Are offered at accredited tertiary institutions

In the event one nursing programme and one medical programme were selected from each of four participating institutions.

#### Exclusion criteria

- Undergraduate Medical and Nursing Programmes in countries outside Eastern and Southern Africa
- Programmes whose students are not available during the short period for the research

#### Sampling

Due to the nature of the inquiry in our study, nonprobability, convenience sampling will be used to apply our judgment when choosing members of the population expected to possess the information on the technology that has been used in health professions education [38]. To obtain a perspective of what the TEHPE situation is in each programme, 8 staff members and 20 students per programme will be conveniently sampled. In each programme someone who is well acquainted with the programme identifies staff members and students who represent the totality of those populations fairly – e.g. (for students) considering gender and year of study. In detail:

- In each programme a person who functions in the capacity of programme director was selected by researchers from that institution, drawing on the local knowledge they have of available staff.
- To select student candidates random sampling was intended to be used, stratified by year of study and gender. From each programme four students, two male and two female, would be selected randomly from the list of students in each year of study – so totaling 16 students for a four-year programme, 20 for a five-year programme, and 24 for a six-year programme.
- From each programme six educators would be sampled purposively to obtain a spread of candidates by seniority, gender and academic discipline, as follows:
- Seniority: 3 lecturers, 3 senior lecturer/professor
- Gender: 3 male, 3 female

### Data collection procedures

An online questionnaire (using Microsoft Forms) with closed and open-ended questions obtaining quantitative and qualitative data respectively was made available to the programme directors who identified eligible staff members and 20 students with an equal representation of the years of the programme e.g. 4 students per year in a 5-year programme. These were educators and students who were available and willing to respond to the online questionnaire. All data were entered into the Microsoft Forms database set up for the research which was maintained at the University of Botswana. The questionnaires for programme directors, students and educators are appended (Appendices 1, 2 and 3).

### Data analysis and presentation

#### Quantitative data

- These data were summarized using frequency distributions and averages, using appropriate tables.
- Associations and correlations between the data collected for Objectives 1–6 (dependent variables) and biographical and institutional data (independent variables) could not be determined since random or full population sampling was not done.

#### Qualitative data

These data were analyzed thematically (display, reduction, concluding, verifying) using the 6 study objectives as a guide to identifying themes. However, the researchers carried out the first step of the analysis by using ChatGPT with appropriate prompts and then checked the result against the raw data.

This process involved the following steps:

- The raw data was distributed to each individual author for familiarization
- ChatGPT generated the first set of themes
- The themes from ChatGPT were shared with each individual author for review. This was a validation process where authors checked the themes for accuracy and comprehensiveness against the raw data. Authors were expected to verify that the themes were supported with evidence from the data or suggest more appropriate themes if necessary.
- All authors approved the themes

#### Analysis using the SAMR framework

Findings from quantitative and qualitative data analysis were partly organized according to the SAMR framework to portray the types of technology in use and challenges encountered.

### Limiting bias

Selection/ sampling bias: we chose only to use nursing programmes offering a bachelor’s degree in a university. The great majority of nursing programmes are at diploma level, and we therefore failed to gather information about these relatively under-resourced programmes.

### Pilot study

The instrument (whether validated or not) was pre-tested using 1 programme directors, 4 students and 3 educators at the University of Botswana. These persons were not included in the sample. Corrections were made as indicated.

### Study limitations

The main study limitation was the convenience sample and small size which may not be fully representative of the population. This can be rectified in a subsequent study, using stronger probability sampling methods with larger samples or by conducting case studies to give qualitative depth to the main findings.

## FINDINGS

### A. The population

Four institutions in three countries took part in the study: the University of Botswana, the University of Zimbabwe, the National University of Science and Technology (also in Zimbabwe), and the University of Zambia. In each institution the undergraduate medical and nursing programmes too part – so eight programmes in all. The University of the Free State was intending to take part but was unable to do so due to delays in obtaining ethical consent for the research.

Three groups of stakeholders were included in the study population in each programme: programme directors, educators and students. According to the study protocol the sample in each programme was due to include one programme director, six educators, and four students in each year of study. For various reasons this target was not adhered to in most cases. These discrepancies can probably be attributed to the fact that the research had to be expedited to meet a given deadline.

One program director did not respond. The term program director proved somewhat ambiguous, which is why programmes included from one to fi veindividuals in this category, depending on their circumstances. Positions fi lled included professor (1), senior lecturer (4), lecturer (4), assistant dean (1), headof department (2), programme coordinator (1), consultant physician (1) and epidemiologist/ biostatistician (1). Only two had specifi c roles in educational technology; other roles fulfi lled included research, policy design, review of regulations, timetabling, community service and clinical duties. Only three had hadtraining in educational technology.

Medical educators outnumbered nursing educators. In some cases, medical educators taking part did not work with more junior students. The Educatorsunderwent a variety of training events in educational technology, ranging from self-learning to structured formal courses and workshops, with a focus onplatforms like Moodle, Microsoft Teams, and Redcap. There was an emphasis on practical demonstrations and hands-on training, often provided by theuniversity’s library, ICT department, and external educational institutions. While most Educators have some training, there is variation in the extent andcoverage of the training they received.

In the majority of programmes, the number of students sampled considerably exceeded the number stipulated in the research protocol. The number ofmedical student respondents also greatly exceeded nursing students, and in many the distribution according to year of study was unequal.

### B. The types of educational technology being used

All three groups of respondents were asked to report on the types of technology they use in their programmes. They were asked to select from the following list:

1. Online learning platforms (e.g. Moodle, Blackboard, Google Classroom)
2. Online Anatomy, Histology and Pathology resources
3. Online meeting platforms (e.g. Zoom, Teams)
4. Online information retrieval (e.g. PubMed, HINARI)
5. Online document creation (e.g. OneDrive, GoogleDocs)
6. Data collection tools (REDCap etc.)
7. Online tests and exams
8. Social media (e.g. Facebook, WhatsApp, Instagram)
9. Simulations for leaning skills and clinical decision making
10. Evidence-based medical/ health information (e.g. UpToDate, Dynamed, UCentral)
11. Free Open Science resources (e.g. Medscape)
12. Consumer health databases (e.g. MedlinePlus)
13. Artificial Intelligence applications (e.g. ChatGPT, DALL-E)

They were also asked to report on other technologies used.

Other technologies mentioned included Google Meet and WhatsApp Voice Notes.

The averages calculated for all the educators together give an indication of the frequency of use of each technology:

- Most commonly used: online platforms, online meetings, social media, information retrieval, document creation
- Least commonly used: consumer databases, simulations, anatomy apps, online exams,

Other technologies used are reported in A.5 below.

Again, the averages give an indication of the frequency of use of each technology. The pattern corresponds broadly to that of educators with

- Most commonly used: online platforms, online meetings, social media, document creation, AI applications
- Least commonly used: consumer databases, simulations, online exams,
- Of the moderately used group information retrieval is more frequently used by educators and AI applications by students.

Other technologies used are reported in A.5 below.

In programmes with reasonable numbers of educators and students (marked *) there are considerable differences between the two groups in terms of sometechnologies, indicating different uses (and perhaps perceptions of use) by the two groups.

### C. Frequency with which technologies are being used

Respondents were asked to report on the frequency with which different technologies are used in their programmes, as follows: Daily, Weekly, Monthly, Occasionally or Never. For convenience in the analysis these categories were collapsed as follows:

**Table:** 

Daily, Weekly, Monthly	Regularly
Occasionally, Never	Seldom/Never

Again, there is a wide variation in frequency of use. Some general observations:

- Nursing educators report more frequent use of online platforms, online meetings, online exams and simulations than their medical counterparts, but less frequent use of anatomy resources, information retrieval, document creation, information platforms and free science resources.
- Nursing students report more frequent use of simulations than their medical counterparts, but less frequent use of several of the other technologies. Nursing students report less frequent use of online platforms, online meetings, information retrieval, online exams and free science resources than their Educators, but more frequent use of anatomy resources, social media and AI applications
- Medical students report less frequent use of online meetings, information retrieval, online exams and free science resources than their Educators, but more frequent use of anatomy resources, data collection, social media, simulations, information platforms and AI applications

### D. Reasons why these technologies are being used

There is wide variation between levels of respondent, programmes and institutions. When data for all institutions are summarised as in the last two rows in the table above the following appears:

- The Substitution level is scored lowest for both groups of respondents, but higher for the students
- The Augmentation level is scored highest for both groups, but higher for programme directors and educators.
- The Redefinition level is second highest for both groups.

#### D.2 Other reasons given for the use of technology

- Convenience & time efficiency: a very common reason, mentioned by 9 participants, revolves around the convenience and time-saving nature of educational technology.
- Improved learning & understanding: many students and Educators emphasized how technology helps enhance understanding of complex subjects, especially in clinical skills, anatomy, and simulations.
- Large class management: educational technology is often used to manage large class sizes and support distance learning, a theme mentioned several times.
- Access to resources: several mentions about the ability to access a broader pool of educational resources, especially during and after COVID-19.
- Improved clinical skills: simulation-based learning technologies are key for improving clinical practice and skills, frequently highlighted by respondents.
- Global collaboration & communication: technology enables collaboration across distances and time zones, enhancing both student interaction and feedback from instructors.

### E. Stakeholder opinions about the usefulness of the technologies

#### E.1 Usefulness of technology in *administration*: comments from programme directors

Altogether 13 comments were made. In summary:

1. Assessment and tracking:
  - Continuous assessment: technology helps in achieving continuous assessment and tracking student progress.
  - Grade management: technology is used for managing grades and academic records, simplifying administrative tasks related to assessment.
2. Interaction and communication:
  - Online interaction: administrators appreciate technology for enabling communication with students, allowing them to address queries and concerns remotely.
  - Remote learning: during covid-19, online tools were instrumental in continuing education despite physical restrictions.
3. Operational efficiency:
  - Reduced need for physical space: technology reduces the demand for physical space, as learning can be conducted remotely, eliminating the need for booking venues for lectures and seminars.
  - Time efficiency: technology is seen as making teaching more comfortable, timely, and manageable, easing the administrative burden.

#### E.2 Usefulness of technology in *teaching and learning*: comments from programme directors and educators in

Altogether 64 comments were made. In summary:

1. Overall utility:
  - Very useful: technology is regarded as highly beneficial in various aspects, including teaching, learning, and administrative tasks.
  - Effective course delivery: technology is seen as helping to deliver course materials efficiently, aiding both the comprehension of subjects and enhanced engagement between students and instructors.
2. Teaching support:
  - Flexibility and convenience: online platforms, especially during COVID-19, were praised for offering flexibility, allowing teaching to continue seamlessly during disruptions like disease outbreaks. They also reduce travel costs for both Educators and students.
  - One-on-one engagement: online platforms provide more opportunities for personalized learning and student engagement, including the ability to answer students’ queries and support self-directed learning.
3. Assessment and learning efficiency:
  - Continuous and instant assessment: technology has made continuous assessment easier, with platforms offering immediate feedback for students and reducing the administrative burden on educators.
  - Problem-based learning: technologies, such as online quizzes or Socratic methods, facilitate active learning and foster problem-based learning, which is highly encouraged by Educators.
  - Recorded lectures: platforms like Zoom and Teams are particularly useful for recording lectures, enabling students to revisit lessons and revise content, promoting repetitive learning.
4. Access to learning materials:
  - Preparation of teaching materials: technology is seen as very useful for preparing teaching materials, making the process more efficient and timelier.
  - Easy access: educational technology simplifies access to previously taught topics and course materials. Moodle, for instance, is mentioned as a tool regularly used for communication and material distribution.
  - Database utilization: technology also helps educators and students utilize important databases that may have been previously underused.
5. Encouraging self-learning and engagement:
  - Self-directed learning: tools like AI (e.g. ChatGPT) and platforms such as Socrative encourage self-learning and independent research, fostering student-centred learning.
  - Visual aids and interactive learning: audiovisual aids are frequently used to enhance understanding, particularly for visual learners.
6. Student and teacher interaction:
  - Enhanced interaction: technology enhances teacher-student interaction, especially in cases where physical meetings are not possible. The ability to access learning materials remotely and engage online improves the overall learning experience.
  - Diverse learner needs: it helps cater to different types of learners, offering diverse ways to approach the content (e.g. visual, text-based, or interactive).
7. Clinical learning:
  - Clinical skill development: for clinical students, technology can be used to polish skills before they are assigned to real-life settings, especially in terms of simulations and preparatory tasks.
  - While focusing on the usefulness of technologies, respondents also mentioned challenges and limitations:
  - Connectivity issues: despite its benefits, technology’s effectiveness is sometimes hindered by poor internet connectivity and limited access to devices for students.
  - Over-reliance on technology: there are concerns about students relying too much on technology for learning, potentially neglecting important hands-on or real-life learning experiences, especially in clinical settings.
  - Limited use post-COVID: while technology was essential during the pandemic, some instructors noted that its use has reduced after returning to in-person classes, although some platforms (like Zoom or Teams) are still used for lectures.

#### E.3 Usefulness of technology in *teaching and learning*: comments from students in

Altogether 262 comments were made. In summary:

The opinions from both medical and nursing students reflect a strongly positive view of educational technology. Educational technology was regarded as a crucial tool for supporting learning, and numerous benefits were cited, with frequency indicated for each benefit:

1. Ease of access to information (*high frequency*)
  - Students appreciated the convenience of accessing learning materials such as lecture notes, videos, articles, and research papers from anywhere, at any time, which significantly reduced the need for physical resources and travel.
  - Many students mentioned how easy access to resources like UpToDate, Google Meet, YouTube and digital textbooks – all of which helped streamline their learning experience, especially for those dealing with geographic or scheduling challenges.
2. Time and cost savings (*high frequency*)
  - Many students noted the time-saving benefits of using educational technology, particularly by eliminating the need for commuting and enabling access to resources online at their convenience.
  - Students emphasized how online platforms allowed for more efficient learning, with easy access to lectures, readings, and other resources, which also reduced costs associated with physical attendance and materials.
3. Improved understanding and retention (*high frequency*)
  - Visual aids, videos, interactive learning tools, and simulations were praised for helping students better understand complex concepts, particularly in fields like anatomy, pathology, and clinical conditions.
  - Students consistently mentioned how these resources enhanced their comprehension, especially when tackling difficult subjects. Tools like 3D anatomy models and clinical simulations were particularly highlighted as enhancing understanding.
4. Flexibility and convenience (*high frequency*)
  - Students strongly valued the flexibility offered by e-learning platforms, allowing them to study at their own pace and revisit materials as needed. This flexibility was especially important for students balancing clinical placements, work, and personal commitments.
  - A major benefit that technology was seen to be that it facilitated self-paced learning, enabling students to fit their studies around other responsibilities.
5. Increased engagement and collaboration (*high frequency*)
  - Educational technology fostered greater collaboration through online discussions, group meetings, and real-time feedback. This helped improve peer interaction and communication with instructors, which enriched the learning process.
  - Especially in nursing programmes simulation training and group-based digital tools were seen to be crucial for bridging the gap between theory and practice.
6. Access to global resources (*moderate frequency*)
  - Students appreciated the global access to research, new medical practices, and updated guidelines facilitated by technology. This was seen to be especially important in fields like medicine and nursing, where staying up-to-date is critical – so modernising education through technology was an essential development.
  - Students often cited resources like Amboss and UpToDate, as well as online journals, as essential resources that kept them informed about the latest trends and in their fields.
7. Enhanced learning for remote locations (*moderate frequency*)
  - Technology was particularly helpful for students located in remote or rural areas, allowing them to connect with instructors and access learning materials despite geographic/ logistical barriers.
  - While focusing on the usefulness of technologies, respondents also mentioned challenges and limitations:
  - Network and connectivity Issues: many students faced challenges with unstable internet connections, which occasionally disrupted their ability to attend online lectures or access materials efficiently. When sessions used platforms like Google Meet or Zoom, this created significant disruptions.
  - Over-reliance on technology: a few students expressed concerns that over-dependence on technology could diminish the value of hands-on clinical experiences. They felt that virtual learning, while helpful, could not fully replicate the benefits of real-life practice and patient interaction. The need was expressed to balance online learning with practical, in-person training to maintain a well-rounded education.
  - Unequal access to resources: some students noted that unequal access to the necessary technology, such as reliable internet or devices, created disparities in the learning experience. Those without adequate access to technology faced significant barriers, hindering their ability to fully benefit from digital learning tools.
  - Reduced integrity of assessments: a few students raised concerns about the integrity of online assessments, suggesting that remote exams could lead to cheating or less rigorous evaluations.

### F. Obstacles to the use of educational technologies

Obstacles experienced by programme directors and educators differed considerably between institutions and programmes. The differences between nursing and medicine programmes overall were somewhat clearer:

- In nursing programmes student objections, lack of training, poor connectivity and lack of hardware were mentioned more frequently as obstacles.
- In medical programmes staff being unwilling was more frequently cited.

Additional obstacles mentioned included frequent power cuts (3), huge student numbers leading to poor access to some technology which can only handle limited numbers, and data security.

#### F.2 Obstacles to the use of technology experienced by students

Altogether 270 comments were made. In summary the obstacles students face regarding the use of educational technology can be categorized as follows, with frequency indicated for each category:

1. Network and connectivity Issues (*high frequency*)
  - Unreliable or poor internet connection was cited as a major obstacle. Many students report frequent internet issues, including slow speeds, intermittent connections, and weak signals, especially in certain areas (e.g. campus, home, or rural areas).
  - Frequent network breakdowns: problems with WiFi, mobile data, and network outages (including power cuts or load shedding) were commonly mentioned as barriers to online learning.
  - Limited access to WiFi: some students do not have reliable WiFi access, either at home or in other learning environments.
2. Device and equipment limitations (*moderate frequency*)
  - Lack of devices: some students do not have access to personal devices such as laptops, smartphones, or tablets, limiting their ability to engage in online learning.
  - Outdated or malfunctioning equipment: many students report using old, slow, or broken devices that cannot support online learning effectively.
  - Limited access to necessary technology: some students face difficulties due to the unavailability of suitable devices or software at home or in the campus environment.
3. Financial constraints (*moderate frequency*)
  - Cost of internet/data bundles: several students mentioned that the cost of consistent data usage for accessing educational technology is a major obstacle. Some cannot afford the daily or weekly bundles required for learning.
  - Subscription fees for tools or resources: certain educational platforms or resources (e.g., databases, software like UpToDate) require subscription fees, which many students cannot afford.
4. Technical and software issues (*moderate frequency*)
  - Technical malfunctions: software glitches, hardware failures, and malfunctioning systems (e.g. video conferencing tools like Zoom) disrupt the learning process.
  - Complicated platforms: some students reported difficulties with navigating or using certain platforms effectively due to lack of training or familiarity.
  - Limited access to platforms: some tools or platforms used for education (like Zoom) have limitations on the number of participants, preventing large classes from joining.
5. Lack of skills or training (*moderate frequency*)
  - Limited technical knowledge: some students feel they lack the skills necessary to use digital platforms and educational technology effectively. They may struggle with navigating tools or understanding how to use them fully.
  - Need for more training: many students expressed a desire for better training on how to use the available educational technologies to maximize their learning experience.
6. Distractions and decreased focus (*moderate frequency*)
  - Distractions from non-educational content: with online learning, some students get distracted by social media, entertainment, or unrelated websites, leading to wasted time.
  - Reduced attention span: extended screen time or the nature of online learning can contribute to fatigue and reduced ability to focus, leading to poor engagement in lessons.
  - Lack of social interaction: online learning limits face-to-face interactions, reducing the opportunities for peer collaboration and social learning.
7. Power interruptions and load shedding (*moderate frequency*)
  - Frequent power interruptions, particularly in countries with unreliable electricity supply, hinder the ability to attend online classes or access digital materials.
8. Content and curriculum issues (*low frequency*)
  - Some students find that the information available online doesn’t always align with practical experiences, making it hard to reconcile what they learn with what they encounter in clinical settings.
  - Outdated information: some educational resources are outdated or less relevant, causing students to question their usefulness for current learning needs.
9. Limited lecturer support (*low frequency*)
  - Some students noted that lecturers are not always motivated or equipped to use educational technologies effectively, which impacts their ability to learn from digital platforms.
10. Health and ergonomics issues (*low frequency*)
  - Prolonged use of electronic gadgets without breaks can lead to physical strain, such as repetitive strain injuries, affecting students’ health and ability to continue learning effectively.
11. General frustrations (*low frequency*)
  - Overload of digital content: managing multiple online platforms and sources of information can be overwhelming for some students, leading to confusion and inefficiency in accessing materials.
  - The specific descriptions of obstacles are a helpful addition in understanding the quantitative evaluations of educators and programme directors above.

## DISCUSSION AND CONCLUSIONS

### General comments

The research intended to produce information concerning TEHPE in sub-Saharan Africa, but in the event only covered three countries, four institutions and eight medical and nursing programmes. One other institution (University of the Free State) was ready to participate but was held back by delays in its local ethical review process. The research process and methodology were also not ideal: numbers of respondents varied widely (Tables 4, 5 and 6) and were not in accordance with the samples stipulated in the protocol (pp.8–9 above). The decision was therefore taken to use all the data provided and not to limit the number of respondents to those stipulated in the protocol. Random sampling was mostly not done so that statistical comparisons could not take place. These defects can mostly be attributed to the strict timeline for the research as well as the multiplicity of sites and programmes, which had a negative effect on communication between research partners. The research design was probably over-ambitious, considering the time available to carry it out. The short timeline also affected the analysis of the very large amount of qualitative data produced, so that the research team decided to use an AI application for much of the analysis (briefly checked against the raw data afterwards). The short timeline also affected our ability to compare our findings in detail with those of other studies – ideally, we should also have done a scoping review of TEHPE studies in Africa and elsewhere, but this is a task that remains to be done in future. Ideally it would also have been useful to know what the situation was concerning TEHPE before the COVID pandemic struck. However, in spite of these methodological and other deficiencies the research produced a large amount of information concerning TEHPE in the three countries, four institutions and eight medical and nursing programmes. As far as we know this interesting information in such detail was not available before and opens up many opportunities for further research as well as for planning and implementing improvements in TEHPE. Individual programmes and institutions can usefully compare themselves with these eight programmes.

In the remainder of this section the findings will be discussed in relation to each of the research objectives, as follows:

1. Determine the types of educational technology being used
2. Identify the frequency with which different technologies are being used
3. Reveal the reasons why these technologies are being used
4. Determine teacher and student opinions about the usefulness of the technologies
5. Identify obstacles to the use of these technologies
6. Verify factors facilitating the use of these technologies

Note that data collection for Objective 6 was omitted since it was found that the sections in the instruments providing data for it simply mirrored the ones for Objective 5.

### Objectives 1 and 2: The types of educational technology being used and how frequently (Tables 7, 8, 9, 10, 11, 12)

#### Reported use of technology

The 13 types of TEHPE listed were drawn from the literature and the individual experiences of the researchers. The findings indicate that this list was probably sufficient, since ‘Other technologies used’ (Tabe 11) were relatively few – only 89 instances were mentioned by 299 respondents, and of these 89 some were basic hardware (laptops, data projectors) and online data source, which could fall under one or more of the 13 types.

The numerical analysis of data was the first step in the analysis, with percentages being used to compare institutions and programmes (statistical comparisons were not possible, as explained above). The numerical analysis showed extreme variation (between programmes and institutions) in the use of technologies. This finding is best explained by variations in the way in which programmes are offered and ‘educator’ preferences – there is clearly no ‘one size fits all’.

Evaluation of the data in each table was undertaken as a second step of analysis, with the main findings given in the text below each table. Highlights:

- Technology is being used extensively in these programmes, with ‘online exams and ‘consumer databases’ somewhat less than the others.
- The ‘programme director’ responses were few and did not add significant information (Table 7). This category could probably have been left out of the study since it turned out that many of these persons were also ‘educators’.
- For the ‘educators’ (Table 8) the technologies most used were online platforms, online meetings, social media, information retrieval and document creation. Less used were consumer databases, simulations, anatomy/histology apps, and online exams.
- For the ‘student’ group (Table 9) the most used technologies were online platforms, online meetings, social media, information retrieval and document creation. Less commonly used were consumer databases, simulations, anatomy/histology apps and online exams.
- Of the moderately used group information retrieval is more frequently used by educators (to be expected in view of the research tasks of educators) and AI applications by students (probably to be expected since students are ‘technology natives’).
- Generally, NUST educators and students report less use of technology than the other three institutions (Zimbabwe nursing having to few data to compare). The reasons are not clear and could indicate new research.
- Comparisons between student and educator use (Table 10) was difficult to do in view of several programmes lacking enough responses to compare – in fact only three programmes could be analysed in this way. In the table below these three are compared (pink shading: educators use more; blue shading: students use more):
- The findings again illustrate the considerable variability between programmes and institutions. However, some findings stand out (for these three programmes):
- Students use more Anatomy/Histology, Information and AI resources
- Educators use more information retrieval resources.

#### Frequency with which technologies are used

The frequency with which the different technologies are being used was also determined (Table 13). Again, there is a wide variation in reported frequency of use. Some general observations:

- Nursing educators report more frequent use of online platforms, online meetings, online exams and simulations than their medical counterparts, but less frequent use of anatomy resources, information retrieval, document creation, information platforms and free science resources.
- Nursing students report more frequent use of simulations than their medical counterparts, but less frequent use of several of the other technologies.
- Nursing students report less frequent use of online platforms, online meetings, information retrieval, online exams and free science resources than their Educators, but more frequent use of anatomy resources, social media and AI applications
- Medical students report less frequent use of online meetings, information retrieval, online exams and free science resources than their Educators, but more frequent use of anatomy resources, data collection, social media, simulations, information platforms and AI applications

The inevitable conclusion is that technology is a ‘movable feast’, used in different ways by educators and students to meet their individual needs in teaching and learning.

Comparison of reported use and frequency of such use (Table 16, Appendix 5) showed that, in general, reported use and frequency of use harmonised. In some cases (as expected) reported use of a technology exceeded the regularity of its use. Interestingly however reported regular use of a technology exceeded its reported use in several cases (highlighted in pink in Tabe 16). This could be due to ‘questionnaire fatigue’ or (which is more likely) respondents being stimulated by the ‘frequency’ question to dig more deeply into their memories.

#### Technology needed but not available

Information was also provided about technologies that are needed but not available (Table 12). Some of the 112 requests indicate shortages in the 13 technologies already listed (e.g. simulations, online resources for anatomy, AI applications); some that concern hardware (e.g. personal devices, visual display equipment); and others go beyond that (e.g. virtual reality). Some of these needs can be met without cost, but others need funding and may not be so easily met.

### Objective 3: Reasons why technologies are being used

#### Reasons given according to the SAMR categories (Table 14)

As set out in the Conceptual Framework (pp. 4–5) the four categories are classed as follows:

- **Enhancement** – Substitution and Augmentation

Digital technology is substituted for analogue technology, but without functional change

- **Transformation** – Modification and Redefinition

Technology enables significant task redesign and the creation of previously inconceivable tasks.

There is wide variation between categories of respondent, programmes and institutions. This variation is to some degree unexpected since the reasons are not logically linked to types of technology used and rather reflect current practice (or understanding of what practice should ideally be). Respondents were asked to ‘identify the reasons for using educational technology in your programme’; however, answers given may reflect a respondents’ understanding of what is actually being done, but also of what should ideally be done. When data for all institutions are summarised in the table (joining programme directors and educators into one group as ‘Educators’), the following:

- The Substitution level is scored lowest for both groups of respondents. This is an encouraging development if it truly reflects the reality of current practice. The fact that students experience more at this level may well indicate that the ‘teacher’ group knows that higher levels of SAMR are desirable but sometimes fall back onto Substitution (which is then what the students experience).
- The Augmentation level is scored highest for both groups, but higher for ‘Educators’. This may well reflect current practice – technology enables rapid change at this level but is more difficult to achieve at the higher levels.
- The Modification and Redefinition levels are strongly represented – in fact the Redefinition level is second highest for both groups. This is an important finding, indicating that technology is substantially being used (in reality by Educators and students, or in Educators’ intentions for future action).

#### Other reasons given for using technology

These are summarised in six categories in D.2 above. A key concept underlying some of these categories is the support that technology gives to time management, teaching large classes, access to resources, and student-educator communication. Educational support is also mentioned: technology assisting the understanding of complex topics and subjects and improving clinical skills through simulations. These reasons could usefully be explored further by additional research.

#### Links between technology and the SAMR classification

In Table 17 an intuitive effort is made to link the kind of technology used with the SAMR categories in mind. Some technologies are clearly linked to the higher (MR) or lower (SA) levels, but formal research will be needed to clarify which technologies strongly support each of the levels. Once those which support the higher (MR) levels have been clarified, steps can be taken to implement them more rigorously.

### Objective 4: Teacher and student opinions about the usefulness of technology

#### Usefulness of technology in programme administration

Programme directors’ observations are summarised in E.1 above. They focus on practical issues such as managing student records, improved interaction and communication with students, and operational efficiency (e.g. economical use of space and time). These administrative benefits do not come to mind as much as the educational ones do, but they are real and of considerable benefit to managers.

#### Usefulness of technology in teaching and learning (for ‘Educators’ and ‘learners’)

These observations are summarised in E.2 and E.3 above. For both groups the engagement with the question of ‘usefulness’ has been remarkable: the 47 ‘teacher’ respondents made 64 comments, and the 264 students made 262 comments. A wealth of raw data is available which deserves more attention than that given to the brief summaries above.

The content of comments within a respondent group sometimes overlapped, but less so between respondent groups, as can be seen in Table 18:

From the table the benefits for ‘Educators’ were those which support them in their role as Educators/ facilitators, with some thought also given to the usefulness of technology for students. The students on the other hand (unsurprisingly) focused on how technology was supporting their learning. Although respondents were asked to list the benefits they perceived technology to offer, they also used the opportunity to mention challenges and limitations to its use. These naturally came to mind as reasons for limiting the potential of the stated benefits. As expected, connectivity issues which limit access and learning were mentioned by both ‘Educators’ and students – so too over-reliance on technology which was limiting hands-on skills learning and clinical experience (the technology needed during the COVID pandemic continued to be used at the expense of necessary in-person teaching). Students also noted that unequal access to some technologies, due to the expense involved, was affecting the student body.

### Objective 5: Obstacles to the use of technology

#### Obstacles encountered by programme directors and educators in the use of technology

The obstacles listed in the instruments for programme directors and educators were drawn from the literature and the individual experiences of the researchers. The findings are given in Table 15. Again (as has been the case for technologies used, their frequency of use, and reasons for using them) the obstacles experienced differed considerably between institutions and programmes. As explained before this is not surprising: programmes, Educators and situations differ in many respects, so technology is put to use in situation-specific ways: there is no ‘one size fits all’, also in terms of the obstacles encountered.

The main obstacles identified for all the programmes together include: staff and students lack the required skills; no training is available; poor connectivity; lack of hardware and of funds to buy software; and lack of online learning resources. When summarised the differences between nursing and medicine programmes overall were somewhat clearer:

- In nursing programmes student objections, lack of training, poor connectivity and lack of hardware were mentioned more frequently as obstacles.
- In medical programmes staff being unwilling was more frequently cited.

The obstacles listed in the instruments seem to have been comprehensive, since only a few additional obstacles were mentioned: frequent power cuts (3); huge student numbers leading to poor access to some technology which can only handle limited numbers; and data security.

#### Obstacles encountered by students

These are summarised in F.2 above. In contract to the instrument for ‘Educators’ the students responded to an open-ended question, asking them to ‘describe the challenges you face regarding the use of educational technology.’ Altogether 270 comments were made – again an indication of how seriously the students take the issue of technology in their learning. The specific descriptions of obstacles are a helpful addition in understanding the quantitative evaluations of educators and programme directors above. In summary the main obstacles students face can be categorized as follows. It is important to note how obstacles reported by students coincide with those identified by ‘Educators’ (Table 19):

Students’ specific descriptions of obstacles are a helpful addition in understanding the quantitative evaluations of  Educators’ in Table 15. Additional obstacles specifically related to student needs and which have no ‘Educator’ counterpart are listed above.

##### In summary:

The five research objectives were largely achieved, with large amounts of data being generated in the process. In several cases the findings open possibilities for additional data analysis. As indicated above the findings also indicate enticing avenues for further research on aspects of TEHPE, both in the current institutions and programmes and more widely afield.

## RECOMMENDATIONS

Based on the needs assessment conducted for technology-enhanced health professions education (TEHPE), the following organized recommendations are proposed:

### 1. Infrastructure Development

- Invest in Connectivity by improving internet access in educational institutions, particularly in rural areas. Partner with government agencies and private sector providers to expand broadband coverage and provide affordable data plans for students and educators.
- Access to Devices through implementing programs to provide students and educators with essential devices (laptops, tablets). Consider device-sharing schemes, subsidies, or donations to bridge the hardware gap.
- Reliable Power Supply by advocating for addressing issues of power outages by investing in backup solutions such as solar panels, generators, and uninterruptible power supplies (UPS).

### 2. Training and Capacity Building

- Comprehensive Training Programs by developing and implementing regular training sessions for educators and students on how to effectively use educational technologies. Focus on both basic tools and advanced applications like simulations and AI-driven platforms.
- Digital Literacy Initiatives by inculcating digital literacy into the curriculum to ensure students are proficient in using educational technologies. Offer workshops on navigating platforms, data security, and best practices for online learning.

### 3. Curriculum reviews to align with technology

- Moving towards Higher-Order SAMR Levels by fostering the use of technology at the modification and redefinition levels. For example, integrate simulations for clinical training, virtual reality for anatomy lessons, and AI applications for personalized learning.
- Blended Learning Models by adopting blended learning approaches that combine face-to-face instruction with online tools. This can enhance flexibility, engagement, and access to resources.

### 4. Institutional Support and Policy Development

- Create specialized eLearning Units by establishing eLearning units within institutions to support educators in adopting and integrating technology into their teaching. These units can offer technical assistance, training, and resource development.
- Develop TEHPE Policies by designing clear policies and guidelines for the integration of technology in health professions education. Ensure these policies address equity, accessibility, and sustainability.
- Continuous Monitoring and Evaluation by developing quality assurance mechanisms for ongoing evaluation of TEHPE initiatives. Gather feedback from stakeholders to identify areas for improvement and ensure that technology use aligns with educational goals.

### 5. Collaboration and Resource Sharing

- Regional Partnerships through fostering collaborations among institutions within Eastern and Southern Africa to share resources, best practices, and technological solutions. Establish a consortium or network for technology-enhanced education.
- Open Educational Resources (OER) by promoting the use and development of open-access educational resources to reduce costs and improve access to quality materials.

### 6. Addressing Equity and Inclusion

- Bridge the Digital Divide through implementing targeted interventions to support under-resourced programs and marginalized groups. Ensuring that gender, socio-economic, and geographic disparities are addressed through inclusive policies and practices.
- Support for Students with Disabilities by advocating for and ensuring that educational technologies are accessible to all students, including those with disabilities. Provide assistive technologies and inclusive learning platforms.

## Figures and Tables

**Table 1.** Definition of terms

*Term*	*Definition*
Educational technology	Includes the following: online information retrieval (e.g. PubMed, HINARI); sources of evidence-based medical/ health information (UpToDate, Dynamed, UCentral etc.); free Open Science resources (e.g. Medscape); consumer health databases (e.g. MedlinePlus); social media (Facebook, WhatsApp, Instagram etc.); visual platforms (e.g. YouTube); AI applications (e.g. ChatGPT, DALL-E); online meeting platforms (Zoom, Teams etc.); online document creation (OneDrive, GoogleDocs etc.)
Health professions education	Training of health professionals in formally recognized programmes

**Table 2.** Data to be collected

*Research objectives*	*The information needed to achieve it*	*Data type*
1. Determine the types of educational technology being used	A list of types of educational technology used	Nominal
2. Identify the frequency with which different technologies are being used	For each type of technology being used a measure of the frequency of its use	Ordinal
3. Reveal the reasons why these technologies are being used	For each type of technology being used: • The reason(s) for its use • The circumstances under which it is used	Nominal
4. Determine teacher and student opinions about the usefulness of the technologies	For each type of technology being used, staff and student opinions of: • How useful it is • Reasons why it is useful (or not)	Ordinal Nominal
5. Identify obstacles to the use of these technologies	A list of obstacles to the use of technology, graded for severity	Nominal Ordinal
6. Verify factors facilitating the use of these technologies	A list of factors facilitating the use of technology, graded for helpfulness	Nominal Ordinal
Biographical details of the respondents and variables defining their institutions	• Age, gender, qualifications of respondent • Nature of institution (university, other) • HPE programmes the institutions offers	Interval Nominal

**Table 3.** Nature and location of programmes to be sampled

Programmes in institutions linked to PACoTEH members	Higher education institutions offering health professions education programmes for doctors and nurses at the following participating institutions of higher education: University of Botswana, National University of Science and Technology (Zimbabwe), University of Zambia, University of Zimbabwe

**Table 4.** Programme directors

Institution	Programme	Number	Qualification		Roles	
			Masters	Doctoral	Education/ teaching and learning	Use of specific technology
Botswana	Nursing	0				
	Medicine	1		1	1	
NUST	Nursing	1		1	1	1
	Medicine	1	1		1	
Zambia	Nursing	2	1	1	2	1
	Medicine	5	3	2	5	
Zimbabwe	Nursing	1	1		1	
	Medicine	1	1		1	

**Table 5.** Educators

Institution	Programme	Total no. of educators	Level of students taught										Training in T of E	
			Year 1		Year 2		Year 3		Year 4		Year 5/6			
			N	%	N	%	N	%	N	%	N	%	N	%
Botswana	Nursing	5	4	80.0	5	100.0	3	60.0	4	80.0	1	20.0	5	100.0
	Medicine	6	3	50.0	4	66.7	3	50.0	1	16.7	3	50.0	3	50.0
NUST	Nursing	2	1	50.0	2	100.0	2	100.0	1	50.0	0	0	0	0
	Medicine	8	2	25.0	2	25.0	4	50.0	4	50.0	4	50.0	3	37.5
Zambia	Nursing	3	2	66.7	3	100.0	3	100.0	3	100.0	1	33.3	1	33.3
	Medicine	7	0	0	4	57.1	4	57.1	1	14.3	3	42.9	2	28.6
Zimbabwe	Nursing	1	1	100.0	1	100.0	1	100.0	1	100.0	0	0	0	0
	Medicine	3	0	0	0	0	0	0	1	33.3	3	100.0	0	0
TOTAL	Nursing	11		74.2		100.0		90.0		82.5		13.3	6	54.5
	Medicine	24		18.8		37.2		39.3		28.6		60.7	8	25.0

**Table 6.** Students

Institution	Programme	Total no. of students	Year of study									
			1		2		3		4		5/6	
			N	%	N	%	N	%	N	%	N	%
Botswana	Nursing	9	0	0.0	5	55.6	3	33.3	1	11.1	0	0.0
	Medicine	20	4	20.0	4	20.0	4	20.0	5	25.0	3	15.0
NUST	Nursing	9	8	88.9	1	11.1	0	0.0	0	0.0	0	0.0
	Medicine	54	10	18.5	12	22.2	12	22.2	10	18.5	10	18.5
Zambia	Nursing	29	3	10.3	18	62.1	3	10.3	4	13.8	1	3.4
	Medicine	47	0	0.0	0	0.0	12	25.5	18	38.3	17	36.2
Zimbabwe	Nursing	38	0	0.0	16	42.1	12	31.6	10	26.3	0	0.0
	Medicine	58	7	12.1	12	20.7	4	6.9	10	17.2	25	43.1
TOTAL	Nursing	85	11	12.9	40	47.1	18	21.2	15	17.6	1	1.2
	Medicine	179	21	11.7	28	15.6	32	17.9	43	24.0	55	30.7

**Table 7:** Types of educational technology being used by Programme Directors

Institution	Programme (N)	Kinds of technology used												
		1. Online platforms (Moodle etc.)	2. Anatomy, Histology, Pathology resources	3. Online meetings (Zoom etc.)	4. Info. retrieval (PubMed etc.)	5. Document creation (GoogleDocs etc.)	6. Data collection tools (REDCap etc.)	7. Online exams	8. Social media (Facebook etc.)	9. Simulations (for skills, decision making)	10. Info. platforms (UpToDate, UCentral etc.)	11. Free science resources (Medscape etc.)	12. Consumer databases (Medline Plus etc.)	13. AI applications (ChatGPT, DALL-E etc.)
Botswana	Nursing (N=0)													
	Medicine (N=1)	1	1	1	1	1	1	1	1	1	1	1	1	1
NUST	Nursing (N=1)	1		1	1		1		1					
	Medicine (N=1)	1		1	1	1			1		1	1		
Zambia	Nursing (N=2)	2		2	2	2	2	1	2	1			1	
	Medicine (N=5)	5	1	5	5	4	2	3	5		2	1		3
Zimbabwe	Nursing (N=1)	1		1	1				1					
	Medicine (N=1)			1	1		1		1		1	1		

**Table 8:** Types of educational technology being used by Educators

Institution	Programme (N)	Kinds of technology used (N and %)																									
		1. Online platforms (Moodle etc.)		2. Anatomy, Histology, Pathology resources		3. Online meetings (Zoom etc.)		4. Info. retrieval (PubMed etc.)		5. Document creation (GoogleDocs etc.)		6. Data collection tools (REDCap etc.)		7. Online exams		8. Social media (Facebook etc.)		9. Simulations (for skills, decision making)		10. Info. platforms (UpToDate, UCentral etc.)		11. Free science resources (Medscape etc.)		12. Consumer databases (Medline Plus etc.)		13. AI applications (ChatGPT, DALL-E etc.)	
		N	%	N	%	N	%	N	%	N	%	N	%	N	%	N	%	N	%	N	%	N	%	N	%	N	%
Botswana	Nursing (N=5)	5	100	1	20	5	100	4	80	2	40	2	40	3	60	5	100	3	60	2	40	0	0	0	0	2	40
	Medicine (N=6)	4	67	2	33	6	100	6	100	4	67	3	50	2	33	3	50	0	0	3	50	4	67	1	17	1	17
NUST	Nursing (N=2)	1	50	1	50	1	50	0	0	0	0	0	0	0	0	1	50	0	0	0	0	0	0	0	0	1	50
	Medicine (N=8)	6	75	0	0	3	38	0	0	1	13	0	0	0	0	3	38	0	0	0	0	1	13	0	0	0	0
Zambia	Nursing (N=3)	3	100	1	33	3	100	2	67	3	100	1	33	0	0	3	100	1	33	1	33	2	67	1	33	2	67
	Medicine (N=7)	6	86	1	14	6	86	4	57	5	71	3	43	2	29	3	43	0	0	2	29	2	29	2	29	4	57
Zimbabwe	Nursing (N=1)	1	100	0	0	0	0	0	0	0	0	0	0	0	0	0	0	0	0	0	0	0	0	0	0	0	0
	Medicine (N=3)	3	100	2	67	2	67	2	67	2	67	2	67	1	33	2	67	2	67	2	67	2	67	1	33	2	67
**All educators (N=26)**		**29**	**82.9**	**8**	**22.9**	**26**	**74.3**	**18**	**51.4**	**17**	**48.6**	**11**	**31.4**	**8**	**22.9**	**20**	**57.1**	**6**	**17.1**	**10**	**28.6**	**11**	**31.4**	**5**	**14.3**	**12**	**34.3**

**Table 9:** Types of educational technology being used by Students

Institution	Programme (N)	Kinds of technology used (N and %)																									
		1. Online platforms (Moodle etc.)		2. Anatomy, Histology, Pathology resources		3. Online meetings (Zoom etc.)		4. Info. retrieval (PubMed etc.)		5. Document creation (GoogleDocs etc.)		6. Data collection tools (REDCap etc.)		7. Online exams		8. Social media (Facebook etc.)		9. Simulations (for skills, decision making)		10. Info. platforms (UpToDate, UCentral etc.)		11. Free science resources (Medscape etc.)		12. Consumer databases (Medline Plus etc.)		13. AI applications (ChatGPT, DALL-E etc.)	
		N	%	N	%	N	%	N	%	N	%	N	%	N	%	N	%	N	%	N	%	N	%	N	%	N	%
Botswana	Nursing (N=9)	8	89	2	22	7	78	0	0	5	56	2	22	3	33	6	67	1	11	0	0	0	0	0	0	3	33
	Medicine (N=20)	10	50	15	75	20	100	13	65	14	70	16	80	7	35	10	50	6	30	14	70	13	65	8	40	8	40
NUST	Nursing (N=9)	1	11	0	0	1	11	1	11	2	22	1	11	0	0	9	100	0	0	0	0	1	11	0	0	1	11
	Medicine (N=54)	47	87	5	9	21	39	6	11	10	19	3	6	2	4	10	19	3	6	19	35	13	24	2	4	11	20
Zambia	Nursing (N=29)	24	83	4	14	23	79	3	10	9	31	6	21	1	3	7	24	3	10	5	17	1	3	1	3	5	17
	Medicine (N=47)	46	98	19	40	43	91	14	30	29	62	14	30	14	30	24	51	9	19	20	43	21	45	3	6	21	45
Zimbabwe	Nursing (N=38)	28	74	13	34	27	71	11	29	23	61	9	24	18	47	30	79	20	53	11	29	5	13	9	24	20	53
	Medicine (N=58)	35	60	31	53	53	91	27	47	36	62	18	31	16	28	40	69	9	16	19	33	23	40	10	17	37	64
All students (N=264)		**199**	**75.4**	**89**	**33.7**	**195**	**73.9**	**75**	**28.4**	**128**	**48.5**	**69**	**26.1**	**61**	**23.1**	**136**	**51.5**	**51**	**19.3**	**88**	**33.3**	**77**	**29.2**	**33**	**12.5**	**106**	**40.2**

**Table 10:** Comparison - use of technology by Educators and Students

Institution	Programme	Position	N	Kinds of technology used (%)															
				1. Online platforms (Moodle etc.)		2. Anatomy, Histology, Pathology resources		3. Online meetings (Zoom etc.)		4. Info. retrieval (PubMed etc.)	5. Document creation (GoogleDocs etc.)	6. Data collection tools (REDCap etc.)	7. Online exams	8. Social media (Facebook etc.)	9. Simulations (for skills, decision making)	10. Info. platforms (UpToDate, UCentral etc.)	11. Free science resources (Medscape etc.)	12. Consumer databases (Medline Plus etc.)	13. AI applications (ChatGPT, DALL-E etc.)
Botswana	Nursing	Educators	5	100		20		100		80	40	40	60	100	60	40	0	0	40
		Students	9	89		22		78		0	56	22	33	67	11	0	0	0	33
	Medicine*	Educators	6	67		33		100		100	67	50	33	50	0	50	67	17	17
		Students	20	50		75		100		65	70	80	35	50	30	70	65	40	40
NUST	Nursing	Educators	2	50		20		50		0	0	0	0	50	0	0	0	0	50
		Students	9	11		0		11		11	22	11	0	100	0	0	11	0	11
	Medicine*	Educators	8	75		0		38		0	13	0	0	38	0	0	13	0	0
		Students	54	87		9		39		11	19	6	4	19	6	35	24	4	20
Zambia	Nursing	Educators	3	100		33		100		67	100	33	0	100	33	33	67	33	67
		Students	29	83		14		79		10	31	21	3	24	10	17	3	3	17
	Medicine*	Educators	7	86		14		86		57	71	43	29	43	0	29	29	29	57
		Students	47	98		40		91		30	62	30	30	51	19	43	45	6	45
Zimbabwe	Nursing	Educators	1	100		0		0		0	0	0	0	0	0	0	0	0	0
		Students	38	74		34		71		29	61	24	47	79	53	29	13	24	53
	Medicine	Educators	3	100		67		67		67	67	67	33	67	67	67	67	33	67
		Students	58		60		53		91	47	62	31	28	69	16	33	40	17	64

**Table 11:** Other technologies used by Educators and Students As reported by educators and students these are:

Category	Application/Software
Communication & Collaboration	Google Meet (4) WhatsApp voice notes, Telegram (1 each)
Learning Platforms & Tools	Amboss (7), Ankidroid (2), Nursing apps (3) Med Hub, Teach Me Anatomy, Geeky Medics, SlideShare, Lecturio, Kenhub, Ninja Nerd, Osmosis (1 each)
Research & Libraries	Digital library (9), Google Scholar (3), ResearchGate (2) NCBI, Access Medicine (1 each)
Interactive Learning & Gamification, Virtual & Augmented Reality	Kahoot (3) General Gamification, Virtual reality (1 each) Virtual reality (1)
Quiz & Assessment	Online quiz (2) Zipgrade, Edurole, Online voice recordings (1 each)
Other applications/ software	YouTube (14), MSD (3)
Hardware	Data projector (10), laptop computer (6), whiteboard (4) Camera for online practical (1)

**Table 12:** Technology needed by Educators and Students but not available As reported by educators and students these are:

1. Relating to applications/software:
• Simulations for learning skills, clinical decision making, anatomy (18)
• Online resources for anatomy (14)
• Virtual and augmented reality (14)
• Artificial Intelligence applications (12)
• Online educational resources - EBM, data collection, textbooks, tutorials, exam papers (1)
• Other educational tools - Gamification, Kenhub, Osmosis, Lecturio (9)
• Learning management systems (6)

2. Relating to hardware:
• Personal devices - smartphones, iPads, tablets, laptops (18)
• Visual/display equipment - for virtual reality, projectors, whiteboards, smartboards, digital microscopes (16)
• Simulation equipment (4)

**Table 13:** Frequency with which technologies are being usedby Educators and Students (%)

Position	Programme	Institution	N	1. Online platforms (Moodle etc.)	2. Anatomy, Histology, Pathology resources	3. Online meetings (Zoom etc.)	4. Info. retrieval (PubMed etc.)	5. Document creation (GoogleDocs etc.)	6. Data collection tools (REDCap etc.)	7. Online exams	8. Social media (Facebook etc.)	9. Simulations (for skills, decision making)	10. Info. platforms (UpToDate, UCentral etc.)	11. Free science resources (Medscape etc.)	12. Consumer databases (Medline Plus etc.)	13. AI applications (ChatGPT, DALL-E etc.)	14. Other technologies
Educators	Nursing	Botswana	5	100	0	100	40	40	40	100	80	40	40	40	20	40	60
		NUST	2	100	0	50	50	0	0	0	50	0	0	0	0	0	0
		Zambia	3	100	33	67	33	67	0	0	0	0	0	33	0	0	0
		Zimbabwe	1	100	100	100	0	0	0	0	0	100	0	0	0	0	0
		TOTAL	11	100	18.2	81.8	36.4	36.4	18.2	45.5	45.5	27.3	18.2	27.3	9.1	18.2	27.3
	Medicine	Botswana	6	100	33	100	100	67	33	17	67	0	67	83	17	17	17
		NUST	8	88	25	63	0	13	0	0	37	0	0	0	0	0	0
		Zambia	7	71	14	57	71	86	29	14	43	0	14	43	29	43	0
		Zimbabwe	3	67	33	67	100	67	0	0	67	33	100	100	33	0	0
		TOTAL	24	83.3	25.0	70.8	58.3	54.2	16.7	8.3	50.0	4.2	33.3	45.8	16.7	16.7	4.2
Students	Nursing	Botswana	9	100	56	89	11	56	44	33	89	44	11	11	11	44	22
		NUST	9	22	0	0	0	11	0	0	78	0	0	0	0	0	11
		Zambia	29	55	14	48	7	17	10	3	31	14	21	10	3	21	14
		Zimbabwe	38	34	39	13	32	45	34	24	79	50	26	26	16	61	16
		TOTAL	85	47.1	27.1	31.8	17.7	32.9	23.5	15.3	63.5	31.8	20.0	16.5	9.4	38.8	15.3
	Medicine	Botswana	20	40	65	90	80	75	40	20	65	35	75	85	55	85	45
		NUST	54	91	17	30	11	11	4	4	28	4	30	22	4	2	46
		Zambia	47	70	28	66	40	60	38	17	51	26	38	40	21	38	13
		Zimbabwe	58	48	48	47	41	40	22	17	74	17	28	36	16	47	10
		TOTAL	149	79.2	40.9	61.7	43.6	48.3	27.5	16.1	63.8	20.8	43.6	46.3	21.5	42.3	30.9

**Table 14:** Reasons by Programme Directors, Educators and Students - according to SAMR categories

Institution	Programme	Level		N	Reason for using technology in education							
					Substitution		Augmentation		Modification		Redefinition	
					N	%	N	%	N	%	N	%
Botswana	Nursing	Programme directors		0								
		Educators		5	1	20	4	80	2	40	2	40
		Students		9	3	33	4	44	1	11	4	44
	Medicine	Programme directors		1	1	100	1	100	0	0	1	100
		Educators		6	1	17	6	100	3	50	3	50
		Students		20	1	5	20	100	2	10	11	55
NUST	Nursing	Programme directors		1	0	0	1	100	0	0	1	100
		Educators		2	0	0	2	100	2	100	0	0
		Students		9	0	0	7	78	6	67	7	78
	Medicine	Programme directors		1	0	0	1	100	1	100	1	100
		Educators		8	1	13	6	75	3	38	5	63
		Students		54	16	30	45	83	32	59	41	76
Zambia	Nursing	Programme directors		2	1	50	0	0	2	100	1	50
		Educators		3	0	0	3	100	2	67	3	100
		Students		29	4	14	13	45	7	24	13	45
	Medicine	Programme directors		5	1	20	3	60	5	100	3	60
		Educators		7	0	0	4	57	2	29	4	57
		Students		47	14	30	26	55	15	32	33	70
Zimbabwe	Nursing	Programme directors		1	0	0	1	100	0	0	1	100
		Educators		1	0	0	1	100	0	0	1	100
		Students		38	8	21	25	66	13	34	26	68
	Medicine	Programme directors		1	0	0	1	100	1	100	1	100
		Educators		3	1	33	3	100	2	67	1	33
		Students		58	16	28	42	72	20	34	31	53
AH institutions		Programme directors and educators		47	7	14.9	37	78.7	25	53.2	28	59.6
		Students		264	62	23.5	182	68.9	96	36.4	166	62.9

**Table 15:** Obstacles to the use of technology experienced by Programme Directors and Educators

Institution	Programme	Position (N)	Obstacles to use of technology																			
			1. Staff are unwilling to use		2. Staff lack the skills		3. Students object to the use		4. Students lack the skills		5. No training is available		6. Poor connectivity		7. Lack of hardware (phones, computers)		8. Lack of funds to buy software		9. Lack of online learning resources		10. Institution does not support	
			N	%	N	%	N	%	N	%	N	%	N	%	N	%	N	%	N	%	N	%
Botswana	Nursing	PD																				
		Educ (5)	2	40	3	60	1	20	1	20	2	40	5	100	2	40	2	40	2	40	0	0
	Medicine	PD (1)	0	0	1	100	0	0	1	100	0	0	0	0	0	0	1	100	1	100	0	0
		Educ (6)	3	50	5	83	1	17	2	33	4	67	3	50	4	67	2	33	1	17	0	0
NUST	Nursing	PD (1)	0	0	0	0	0	0	0	0	0	0	1	100	1	100	0	0	0	0	0	0
		Educ (2)	0	0	1	50	0	0	1	50	1	50	2	100	1	50	1	50	1	50	0	0
	Medicine	PD (1)	0	0	1	100	0	0	1	100	0	0	1	100	1	100	1	100	1	100	0	0
		Educ (8)	0	0	1	13	0	0	0	0	1	13	6	75	3	38	4	50	2	25	0	0
Zambia	Nursing	PD (2)	0	0	2	100	0	0	2	100	2	100	2	100	2	100	2	100	2	100	0	0
		Educ (3)	0	0	2	67	2	67	2	67	2	67	3	100	2	67	3	100	2	67	0	0
	Medicine	PD (5)	3	60	5	100	0	0	0	0	3	60	5	100	3	60	5	100	4	80	1	20
		Educ (7)	1	14	5	71	1	14	2	29	3	43	7	100	5	71	6	86	7	100	1	14
Zimbabwe	Nursing	PD (1)	0	0	1	100	0	0	1	100	1	100	1	100	1	100	1	100	1	100	0	0
		Educ (1)	0	0	1	100	0	0	1	100	1	100	1	100	1	100	1	100	1	100	0	0
	Medicine	PD (1)	1	100	1	100	0	0	1	100	1	100	1	100	0	0	1	100	1	100	0	0
		Educ (3)	1	33	3	100	1	33	0	0	2	67	2	67	3	100	1	33	2	67	0	0
ALL	Nursing (15)		2	13	10	67	3	20	8	53	9	60	15	100	10	67	10	67	9	60	0	0
	Medicine (32)		9	28	22	69	3	9	7	22	14	44	25	78	19	59	21	66	19	59	2	6

**Table 16:** Comparison of use of technology by Educators and Students

Institution and programme	Respondent	1. Online platforms	2. Anatomy/Histology	3. Online meetings		4. Info. retrieval	5. Document creation	6. Data collection	7. Online exams	8. Social media	9. Simulations	10. Info. platforms	11. Free science	12. Consumer data	13. AI applications
Botswana Medicine	Educators	67	33	100		100	67	50	33	50	0	50	67	17	17
	Students	50	75	100		65	70	80	35	50	30	70	65	40	40
NUST Medicine	Educators	75	0	38		0	13	0	0	38	0	0	13	0	0
	Students	87	9	39		11	19	6	4	19	6	35	24	4	20
Zambia Medicine	Educators	86	14	86		57	71	43	29	43	0	29	29	29	57
	Students	98	40	91		30	62	30	30	51	19	43	45	6	45

**Table 17:** Link of the kind of technology used with the SAMR categories

*Technology*	*S*	*A*	*M*	*R*
Online learning platforms (e.g. Moodle, Blackboard, Google Classroom)	X	X		
Online Anatomy, Histology and Pathology resources	X	X		
Online meeting platforms (e.g. Zoom, Teams)	X	X		
Online information retrieval (e.g. PubMed, HINARI)		X	X	
Online document creation (e.g. OneDrive, GoogleDocs)			X	X
Data collection tools (REDCap etc.)			X	X
Online tests and exams	X			
Social media (e.g. Facebook, WhatsApp, Instagram)			X	X
Simulations for leaning skills and clinical decision making				X
Evidence-based medical/ health information (e.g. UpToDate, Dynamed)	X	X	X	
Free Open Science resources (e.g. Medscape)	X	X	X	
Consumer health databases (e.g. MedlinePlus)	X	X		
Artificial Intelligence applications (e.g. ChatGPT, DALL-E)			X	X

**Table 18:** comments on usefulness as perceived by Educators and Students

*Usefulness as perceived by ‘Educators’*	*Usefulness as perceived by students*
Overall usefulness for teaching and learning	
Effective course delivery	
Personalized learning	
Preparing learning materials	
PBL method support: available online material	
Support for clinical learning	
Continuous and instant assessment	
Flexibility and convenience	Flexibility and convenience
Access to learning materials	Access to learning materials
Recorded lectures	Access to global resources
Self-learning and engagement	Better learning and retention
Student-teacher interaction	Increased engagement and collaboration
	Learning from remote locations
	Saving time and costs

**Table 19:** Obstacles reported by Students and Educators

*Reported by Students*	*Identified by Educators*
Network and connectivity Issues: unreliable internet connection; network breakdowns: limited Wi-Fi access	Poor connectivity
Power interruptions and load shedding	Frequent power cuts
Device and equipment limitations: lack of devices; malfunctioning equipment; limited access to necessary technology.	Lack of hardware (phones, computers)
Technical and software issues: technical malfunctions disrupt the learning process; complicated platform navigation; limited access to platforms.	
Limited lecturer support: lecturers are not motivated or equipped to use technologies effectively	Staff are unwilling to use technology and lack the skills to do so; training unavailable
Content and curriculum issues: online material may be old or even irrelevant; content overload	Lack of online learning resources
Financial constraints: cost of internet/data bundles; subscription fees for tools or resources	Lack of funds to buy software
Lack of skills or training needed due to limited technical knowledge	Students lack the skills; no training is available
Distractions and decreased focus: distractions from non-educational content; reduced attention span; lack of social interaction	
Health and ergonomics issues: prolonged use of electronic gadgets can affect students’ health	

## Data Availability

The datasets generated during and/or analyzed during the current study are available from the corresponding author on reasonable request

## Abbreviations and acronyms

HPE: Health Professions Education
PACoTEH: Pan-African Consortium for Technology-Enhanced Health Professions Education
SAMR: Substitution, Augmentation, Modification, Redefinition
TEHPE: Technology-Enhanced Health Professions Education
